# Inflammatory infiltrates in parathyroid tumors

**DOI:** 10.1530/EJE-17-0277

**Published:** 2017-08-30

**Authors:** Felix Haglund, Björn M Hallström, Inga-Lena Nilsson, Anders Höög, C Christofer Juhlin, Catharina Larsson

**Affiliations:** 1 Department of Oncology-PathologyKarolinska Institutet, Cancer Center Karolinska (CCK), Karolinska University Hospital, Stockholm, Sweden; 2 Science for Life LaboratoryKTH-Royal Institute of Technology, Stockholm, Sweden; 3 Department of Molecular Medicine and SurgeryKarolinska Institutet, Karolinska University Hospital, Stockholm, Sweden; 4 Department of Breast and Endocrine SurgeryKarolinska University Hospital, Stockholm, Sweden

## Abstract

**Context:**

Inflammatory infiltrates are sometimes present in solid tumors and may be coupled to clinical behavior or etiology. Infectious viruses contribute to tumorigenesis in a significant fraction of human neoplasias.

**Objective:**

Characterize inflammatory infiltrates and possible viral transcription in primary hyperparathyroidism.

**Design:**

From the period 2007 to 2016, a total of 55 parathyroid tumors (51 adenomas and 4 hyperplasias) with prominent inflammatory infiltrates were identified from more than 2000 parathyroid tumors in the pathology archives, and investigated by immunohistochemistry for CD4, CD8, CD20 and CD45 and scored as +0, +1 or +2. Clinicopathological data were compared to 142 parathyroid adenomas without histological evidence of inflammation. Transcriptome sequencing was performed for 13 parathyroid tumors (four inflammatory, 9 non-inflammatory) to identify potential viral transcripts.

**Results:**

Tumors had prominent germinal center-like nodular (+2) lymphocytic infiltrates consisting of T and B lymphocytes (31%) and/or diffuse (+1–2) infiltrates of predominantly CD8+ T lymphocytes (84%). In the majority of cases with adjacent normal parathyroid tissue, the normal rim was unaffected by the inflammatory infiltrates (96%). Presence of inflammatory infiltrates was associated with higher levels of serum-PTH (*P* = 0.007) and oxyphilic differentiation (*P* = 0.002). Co-existent autoimmune disease was observed in 27% of patients with inflammatory infiltrates, which in turn was associated with oxyphilic differentiation (*P* = 0.041). Additionally, prescription of anti-inflammatory drugs was associated with lower serum ionized calcium (*P* = 0.037).

**Conclusions:**

No evidence of virus-like sequences in the parathyroid tumors could be found by transcriptome sequencing, suggesting that other factors may contribute to attract the immune system to the parathyroid tumor tissue.

## Introduction

Primary hyperparathyroidism (pHPT) is a common endocrine disorder presenting as a single parathyroid adenoma, multiglandular disease or rarely as a parathyroid carcinoma. Parathyroid adenomas with histological features suggestive of malignancy without fulfilling the criteria for carcinoma are named atypical adenomas. Genetic studies have revealed recurrent inactivating mutations of the multiple endocrine neoplasia 1 (*MEN1*) gene and the cell division cycle 73 (*CDC73*) gene as causative agents, but other genetic events seem to occur at very low frequencies (1). pHPT frequently present in postmenopausal women and a role for female hormone receptors has been suggested (2, 3). A significant proportion of pHPT remains without a known molecular driver. Patients with chronic renal failure may develop reactive hyperplastic glands (secondary hyperparathyroidism, sHPT) and true clonal tumors (tertiary hyperparathyroidism, tHPT).

Occasionally, histopathological evaluation of parathyroid tumors identifies inflammatory infiltrates. Published cases and autopsy series were recently summarized in a systematic review (4), identifying 69 cases with diffuse lymphocytic infiltrates, 15 cases with parathyroiditis with germinal centers, 6 cases with sarcoidosis, 4 cases with tuberculosis and 2 cases with granulomatous disease. Furthermore, Shi *et al*. applied flow cytometry to characterize cell types in 20 parathyroid adenomas and 5 corresponding normal glands and observed higher but varying levels of tumor CD8+ lymphocytes as compared to the inflammatory cells in the normal glands. The increased CD8+/CD4+ ratio (as compared to peripheral blood) ruled out contamination from adjacent vascular structures, which had previously been suggested as an underlying cause (5).

Studies in tumor virology has revealed prominent prevalence of viral transcripts in several tumor types originating from different organs, e.g. human papillomaviruses in cervical and oropharyngeal squamous cell carcinoma, hepatitis B virus in hepatocellular carcinoma and Epstein-Barr virus in Burkitt’s lymphoma (6). Among endocrine tumors, Merkel-cell carcinomas are characterized by Merkel-cell polyoma virus, and Epstein-Barr virus has been implicated in the progression of papillary thyroid carcinomas (7). While no compelling evidence of a viral background to parathyroid tumorigenesis exists, no study has thoroughly investigated a possible association. Tumor infiltrative lymphocytes has been associated with viral expression in some tumor types (8, 9, 10, 11), but is also observed in solid tumors, which lacks an underlying viral etiology (12, 13).

We hypothesized that (i) presence of tumor inflammatory infiltrates would be associated with a different clinical presentation as compared to tumors without inflammation, (ii) nodal- and diffuse inflammation would be associated with different clinical presentations of parathyroid tumors respectively and (iii) that active transcription of viral sequences could be an etiological factor in pHPT. To investigate the composition of inflammatory infiltrates in parathyroid tumors we screened 10 years of pathology archives at the Karolinska University Hospital (containing more than 2000 parathyroid tumors). We identified and retrieved 55 parathyroid tumors from 53 patients with histological evidence of inflammation. These cases were reviewed and evaluated using immunohistochemistry for cluster of differentiation (CD) markers. Furthermore, a series of tumors was investigated using massive parallel transcriptome sequencing (RNA-seq) and screened for viral-like sequences without prior assumption of viral pathogens (6).

## Methods

### Clinical cases and tissues samples

We searched the pathology archives of Karolinska University Hospital-Solna for the period 2007–2016 for parathyroid tumors presenting with inflammatory infiltrates visible at histological examination. From 2347 parathyroid tumor records, we identified a total of 56 parathyroid tumors with inflammatory infiltrates. After exclusion of a single parathyroid adenoma that presented with manifestation of lymphoma, the remaining 55 tumors were entered into the study on immunohistochemical and clinical characterization. These included 51 adenomas (from 51 patients with a single parathyroid adenoma and pHPT), 2 tumors from a patient with secondary HPT and 2 from a patient with tertiary HPT. Patient files were reviewed for clinical details, biochemical findings, presence of autoimmune diseases and medication at the time of surgery (Supplementary Table 1, see section on supplementary data given at the end of this article). Data of postoperative serum calcium were available for most patients with single adenomas (43/51), and normalization of hypercalcemia was observed in all patients with inflammatory adenoma where this information was available. All parathyroid tumors were classified according to the World Health Organization (WHO) 2004 criteria (14).

Talat *et al.* (4) suggested as criteria for parathyroiditis, ‘interstitial lymphocytes away from the vessels with terminal differentiation and/or formation of germinal centers.’ While all cases with inflammatory infiltrates included in this study fulfilled these criteria, we deliberately avoided using the term parathyroiditis as no cases showed evidence of tumor degeneration and the nomenclature has previously been linked to hypoparathyroidism in type 1 polyglandular autoimmune syndrome (PGA).

For comparison of the 51 inflammatory adenoma cases with tumors lacking inflammatory infiltrates, we used a previously published cohort of 154 parathyroid adenomas (15), with exclusion of atypical adenomas and parathyroid carcinomas. Two adenomas presented with inflammatory infiltrates and were also excluded. The control cohort thus consisted of 142 consecutively collected parathyroid adenomas. Clinical data for adenoma cases with inflammation and the control adenomas without inflammation are presented in Table 1.

**Table 1:** Clinical data for inflammatory pHPT and non-inflammatory pHPT controls.

**Parameter^†^**	Inflammatory pHPT*	Non-inflammatory pHPT**	*P* Value
Cases in cohort, *n*	(51)	(142)	
Tumors in cohort, *n*	(51)	(142)	
Gender (*n* = 51 and 142)			n.s.
Male	14 (27%)	30 (21%)	
Female	37 (73%)	112 (79%)	
Age at diagnosis (*n* = 51 and 142)			n.s.
Median (min–max) (years)	60 (29–84)	61 (30–80)	
Mean (s.d.)	58 (13)	59 (11)	
Diagnosis (*n* = 51 and 142)			
Parathyroid adenoma	51	142	
Tumor weight (*n* = 51 and 137)			n.s.
Median (min–max) (mg)	565 (100–2412)	423 (80–27 800)	
Mean (s.d.) (mg)	732 (556)	936 (2619)	
Predominant celltype (*n* = 51 and 139)			0.002
Chief cell	32 (63%)	98 (70%)	
Oxyphilic	14 (27%)	18 (13%)	
Mixed	5 (10%)	23 (17%)	
Serum ionized calcium (*n* = 46 and 129)			n.s.
Median (min–max) (mmol/L)	1.43 (1.34–1.84)	1.43 (1.31–1.89)	
Mean (s.d.) (mmol/L)	1.46 (0.11)	1.43 (0.07)	
Serum intact PTH (*n* = 49 and 135)			0.007
Median (min–max) (ng/L)	132 (46–342)	109 (56–513)	
Mean (s.d.) (ng/L)	162 (64)	126 (70)	
Plasma phosphate (*n* = 40 and 129)			n.s.
Median (min–max) (mmol/L)	0.82 (0.32–2.1)	0.83 (0.43–1.1)	
Mean (s.d.) (mmol/L)	0.87 (0.18)	0.82 (0.13)	

* Study cohort; **Control cohort; ^†^number of cases with data is indicated within parentheseis for each parameter. Normal reference values for parathyroid gland weight (<60 mg), ionized calcium (1.15–1.33 mmol/L), PTH (10–65 ng/L) and phosphate (0.8–1.5 mmol/L).

*n.s.*, not significant; s.d., standard deviation.

Fresh frozen tumor samples from totally 13 cases were entered into the transcriptome sequencing including: four of the above described 51 adenomas with inflammation and 7 previously published cases without inflammation (16) (Table 2). RNA isolated from fresh-frozen tissue from a Merkel-cell carcinoma was kindly provided by Dr Weng-Onn Lui, Department of Oncology-Pathology, Karolinska Institutet.

**Table 2:** Summary of tumor sequencing information.

**Parathyroid tumor**	**Diagnosis**	**cDNA library generation**	**Seqencing depth** (million reads)	**Sequencing platform**	**Detected virus transcripts**
14	Parathyroid adenoma	RiboZero	50.1	Hiseq 2500	Negative
40	Parathyroid adenoma	RiboZero	51.5	Hiseq 2500	Negative
51	Parathyroid adenoma	RiboZero	60.6	Hiseq 2500	Negative
52	Parathyroid adenoma	RiboZero	58.0	Hiseq 2500	Negative
56	Parathyroid adenoma	TruSeq	229.0	HiSeq 2000	Negative
57	Parathyroid adenoma	TruSeq	215.4	HiSeq 2000	Negative
58	Parathyroid adenoma	TruSeq	220.7	HiSeq 2000	Negative
59	Parathyroid adenoma	TruSeq	234.9	HiSeq 2000	Negative
60	Parathyroid adenoma	TruSeq	134.8	HiSeq 2000	Negative
61	Parathyroid adenoma	TruSeq	226.4	HiSeq 2000	Negative
62	Parathyroid adenoma	TruSeq	188.4	HiSeq 2000	Negative
63	Atypical parathyroid adenoma	TruSeq	231.7	HiSeq 2000	Negative
64	Atypical parathyroid adenoma	TruSeq	196.2	HiSeq 2000	Negative
Control	Merkel cell carcinoma	RiboZero	60.9	Hiseq 2500	Merkel-cell polyomavirus
		TruSeq	53.5	Hiseq 2500	Merkel-cell polyomavirus

### Ethics

All procedures performed in this study involving human participants were in accordance with the ethical standards of the 1964 Helsinki Declaration and its later amendments or comparable ethical standards. Informed consent was obtained from all individual participants included in the study, and ethical permission was granted from the local ethics committee at Karolinska Institutet.

### Immunohistochemistry

All histological slides were reviewed and formalin-fixated paraffin-embedded (FFPE) materials were sectioned for new immunohistochemical analyses using a clinical routine laboratory. Slides were incubated with antibodies for CD4 (4B12, Dako), CD8 (C8/144B, Dako), CD20 (L26, Dako) and CD45 (UCHL1, Dako) all supplied as ready-to-use. After review by two pathologists, two distinct patterns of inflammation were identified; nodular and diffuse. Both patterns were quantified for all CD markers by a pathologist using the following criteria: Diffuse infiltrates were scored as +0 (less than 2% of cells), +1 (2–10% of cells) or +2 (more than 10% of cells). Nodular inflammation was scored as +0 (not present), +1 (single or few small nodular formations) or +2 (several prominent nodules). We observed that both parathyroid normal and tumor tissue sometimes stained positive for CD4 and abstained from scoring the CD4+ lymphocytes in these cases (classified as non-informative in Table 3 and Supplementary Table 2).

**Table 3:** Immunohistochemical analysis of CD markers in inflammatory adenomas.

	**CD4**, *n* (%)	**CD8**, *n* (%)	**CD20**, *n* (%)	**CD45**, *n* (%)
	T helper cells	Cytotoxic T cells	B cells	Leukocytes
Diffuse pattern				
+0 (less than 2% of cells)	12 (60%)	9 (18%)	46 (94%)	7 (14%)
+1 (2–10% of cells)	7 (35%)	26 (51%)	3 (6%)	28 (57%)
+2 (more than 10% of cells)	1 (5%)	16 (31%)	0	14 (29%)
Non-informative*	31	–	–	–
n.a.	0	0	2	2
Nodular pattern				
+0 (not present)	4 (20%)	19 (37%)	11 (22%)	9 (18%)
+1 (single or few small nodular formations)	12 (60%)	26 (51%)	25 (51%)	23 (47%)
+2 (several prominent nodules)	4 (20%)	6 (12%)	13 (27%)	17 (35%)
Non-informative*	31	–	–	–
n.a.	0	0	2	2

* 31 cases exhibited CD4 staining in the parathyroid tissue and scored as non-informative.

*n.a.*, not available (not stained).

### Whole transcriptome shotgun sequencing (RNA-seq)

Fresh-frozen tissues from thirteen parathyroid tumors were collected from the biobank at Karolinska University Hospital, Stockholm, Sweden. Four of these cases were selected among the 51 adenomas based on availability of fresh-frozen tissue. The remaining nine parathyroid tumors (7 adenomas and 2 atypical adenomas) have previously been published as a part of a larger cohort (16). Histological review showed that all specimens were composed of >80% tumor cells. RNA isolated from a Merkel-cell polyoma-virus-positive Merkel-cell carcinoma was used as positive control.

Two rounds of RNA-seq were carried out at SciLifeLab, Karolinska Institutet, Stockholm, Sweden using previously described methodology (17). Total RNA was isolated using the MirVana Isolation Kit (Life Technologies), and tested for integrity (RNA Integrity Number/RIN >8) using an Agilent 2100 Bioanalyzer (Agilent Technologies). For the first round of sequencing, parathyroid tumor cDNA libraries were prepared using the poly-A enrichment based TruSeq cDNA preparation kit, followed by paired-end sequencing to 100 bp on an Illumina HiSeq 2000 instrument (all from Illumina, San Diego, CA, USA). In the second round of sequencing, we aimed to cover potential viral transcripts lacking polyadenylation. Therefore, the cDNA library of the four parathyroid adenomas with lymphocytic infiltrates were generated using a ribosomal RNA depletion method (Ribo-Zero from Illumina) and paired-end sequenced to 125 bp on an Illumina HiSeq 2500 instrument (Illumina). We used a Merkel-cell carcinoma as a positive control, creating cDNA libraries using both Ribo-Zero or TruSeq as described above and sequenced these in parallel with the second batch of parathyroid tumors (on the HiSeq 2500 instrument). All sample sequencing information are summarized in Table 2.

All generated sequencing data (at least 50 million reads per sample) were firstly mapped to the human reference genome (GRCh38) using STAR 2.4.0f (18) in order to sort out any reads originating from human RNA. Remaining reads were mapped to a database of all viral genome sequences in Genbank as of September 2013, using the higher-sensitivity aligner SMALT 0.7.5 (http://sourceforge.net/projects/smalt/), and the number of reads mapping to each viral sequence was recorded. RNA-Seq data from 108 samples of healthy tissues from 31 different organs/tissues (19) sequenced at the same facility was subjected to the same analysis and employed as negative controls to detect technical false positives. As a complement, all generated sequence data were analyzed using the pathogen discovery software READSCAN (20). This method has previously been utilized by Tang *et al*. (6), who successfully identified 28 different known viruses and a novel enterovirus strain among 4433 tumor transcriptomes from 19 different tumor types.

### Statistical analyses

All statistical analyses were performed on IBM SPSS, version 21. Based on distribution of data, non-parametric statistical tests were used, including Mann–Whitney *U* test (for comparison between two groups) and Kruskal–Wallis (for comparison between more than two groups). Relationships between variables were assessed with Spearman’s ranked order correlation. Fisher’s exact test was used for comparison of distribution of categorical variables. All tests were considered two-tailed and a *P* value of <0.05 was taken as statistically significant.

## Results

### Characterization and classification of inflammatory infiltrates

**Figure 1:**
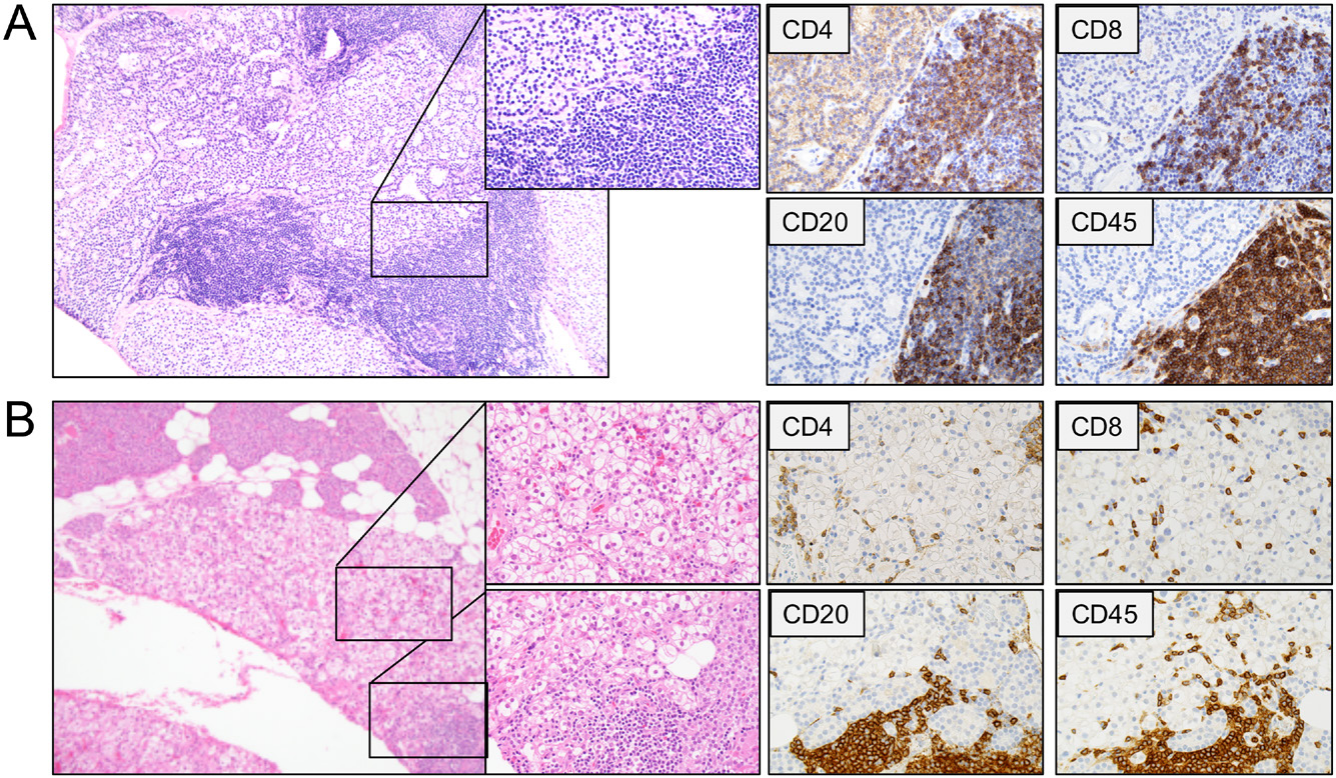
Photomicrographs of routine histology (Htx-eosin, left) and immunohistochemistry (CD4, CD8, CD20 and CD45) of two parathyroid adenomas with (A) mainly nodular (tumor 47) or (B) diffuse and nodular inflammation (tumor 16) respectively. (A) Germinal center-like nodular infiltrates consisted of a mix of CD4+ T-helper, CD8+ cytotoxic T-killer and CD20+ B-lymphocytes. (B) Diffuse tumor inflammatory infiltrates predominantly consisted of CD8+ T-killer cells, but diffuse infiltrates of CD4+ T-helper cells were sometimes observed.

Immunophenotyping of the tumor-infiltrating lymphocytes in 55 parathyroid tumors with inflammatory infiltrates observed at regular histopathology, revealed two distinct patterns. Diffuse inflammations were characterized by lymphocyte infiltrates with CD8+ cytotoxic T-cells and varying levels of CD4+ T-helper cells. For nodular inflammations, germinal center like nodules were observed with CD20+ B lymphocytes, CD4+ T-helper cells and CD8+ T-cytotoxic cells. Examples of diffuse and nodular inflammation are shown in Fig. 1. Cases with nodular formation frequently displayed diffuse inflammatory infiltrates as well. After reviewing the cases, we quantified both diffuse and nodular infiltrates separately for each CD staining (Supplementary Table 2).

**Figure 2:**
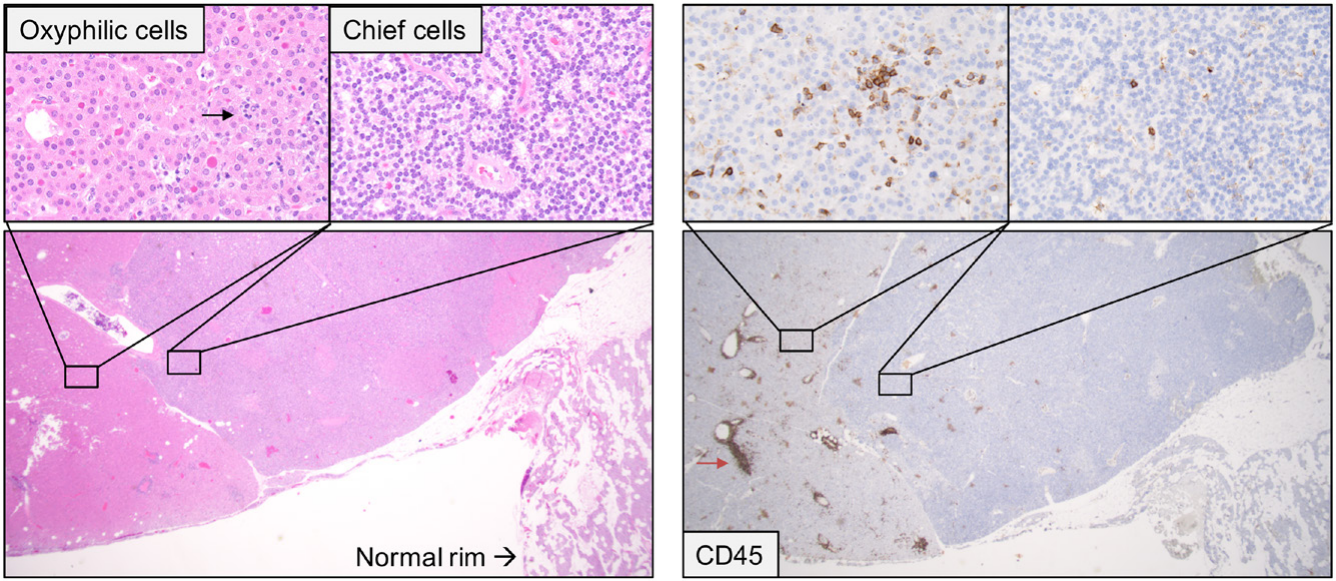
Photomicrographs of routine histology (Htx-eosin, left) and immunohistochemistry for CD45 (right) of a parathyroid adenoma with mixed cell type. Inserts show histologically evident presence of diffusely infiltrating lymphocytes in areas with oxyphilic-(black arrow) but not chief cell differentiation. Immunohistochemical staining for CD45 also revealed presence of lymphocytes in the chief cell areas. There were also prominent perivascular infiltrates of lymphocytes, with one small aggregation of CD20 and CD8+ cells (red arrow, CD8 and CD20 staining not shown).

Among the 51 adenomas a total of 17 cases (33%) had prominent nodular inflammation (+2) and 42 cases (82%) with diffuse inflammation (+1 or +2) as determined by CD45. In 28/51 adenoma cases, we found an adjacent normal rim, which was unaffected by the inflammation in the vast majority of cases (96%). In several adenomas with a mixed cell composition of oxyphilic and chief cells, we observed that the inflammation was predominantly located to the oxyphilic areas (Fig. 2).

Additionally, two patients presented with multi­glandular disease in the context of renal failure. The first patient (sHPT) showed diffuse inflammation in two out of four excised hyperplastic glands. The second patient (tHPT) presented with nodular and diffuse inflammation in two excised adenomatous glands.

None of our 55 tumors exhibited inflammatory mediated degeneration of parathyroid tissue. In our experience, tumor associated stromal fibrosis is observed without relation to inflammatory infiltrates, and the absence of fibrosis in our cohort would argue against a direct link between the two phenomenon.

### Clinical differences between inflammatory and non-inflammatory adenoma cases

**Figure 3:**
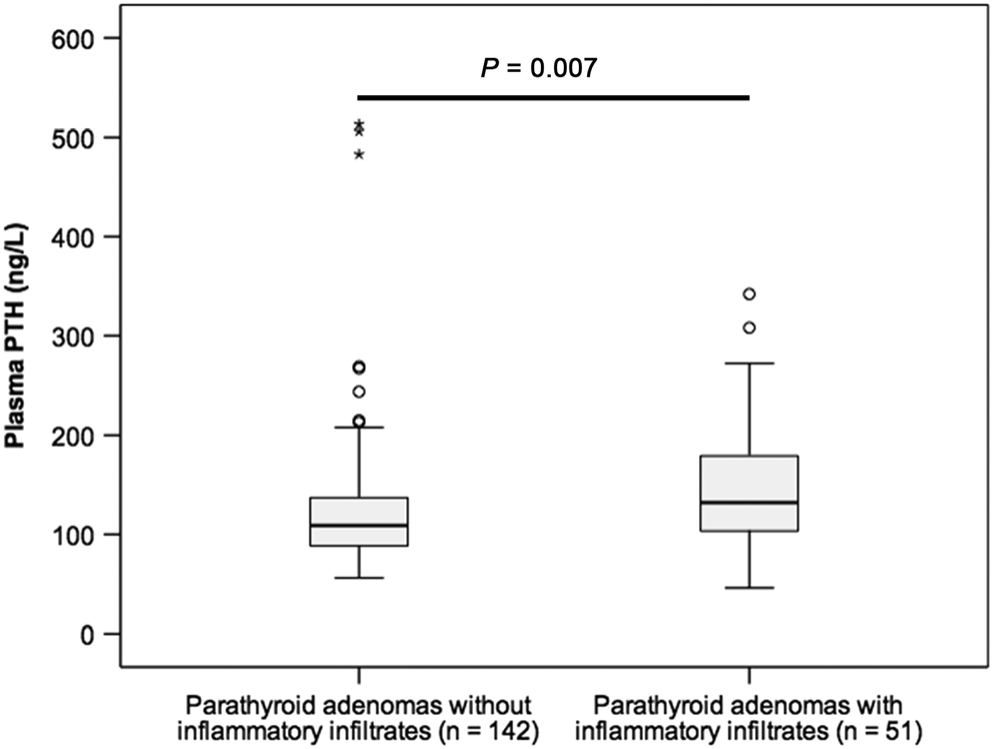
Box-plots of plasma PTH levels in patients with inflammatory parathyroid adenomas (left) and non-inflammatory parathyroid adenoma controls (right). The box plots represent 2nd–3rd quartiles, and whiskers represent the 1st and 4th quartiles. Outliers are represented by open circles and extreme outliers as stars. Parathyroid adenomas with inflammation had significantly higher levels of plasma PTH (Mann–Whitney *U*, *P* = 0.007).

Demographic, pathological and biochemical characteristics were compared between the 51 adenoma cases with inflammatory infiltrates and the control group of 142 adenomas without this feature (Table 1). Inflammatory adenomas were more often of oxyphilic type as compared to the control group (Fisher’s exact test, *P* = 0.002, Table 1). Furthermore, cases with inflammatory infiltrates had significantly higher levels of serum PTH as compared to controls (Mann–Whitney *U* test, *P* = 0.007; Fig. 3), and this association remained significant even after removing the oxyphilic adenomas (*n* = 14) (Mann–Whitney *U* test, *P* = 0.039). No statistically significant differences were observed between inflammatory adenomas and non-inflammatory controls concerning gender, age, tumor weight or patient’s level of serum ionized calcium (including both pre- and postoperative measurements) or plasma phosphate (Table 1).

### Association of inflammatory infiltrates and autoimmune disease

Twelve of 45 patients (27%) with inflammatory parathyroid adenoma had a known autoimmune disease, and a similar proportion (29%; 13/45) was prescribed anti-inflammatory medication at the time of surgery (Supplementary Table 1). Patients with an underlying autoimmune disease more often presented with oxyphilic adenomas (6/13 vs 7/33, Fisher’s exact test, *P* = 0.041). We also observed that anti-inflammatory medication was significantly associated with lower preoperative (but not postoperative) serum ionized calcium (Mann–Whitney *U* test, *P* = 0.037).

The presence of nodular inflammation was not significantly associated to tumor weight, dominating cell type, patients’ levels of PTH, calcium, phosphate, patient age or gender.

### Absence of viral transcripts in parathyroid tumors

Using RNA-seq, we identified Merkel-cell polyoma virus transcripts in both the polyadenylation-enriched (7781 reads) and the ribosomal RNA-depleted cDNA libraries (12 374 reads) of the Merkel-cell carcinoma, thus validating the method to detect viral transcript in this material. No virus transcripts were detected in the cDNA libraries of the 13 parathyroid tumors investigated.

## Discussion

In rare cases of parathyroid tumors, histological analysis reveals infiltrates of inflammatory cells. Inflammatory disorders in other endocrine organs are often associated with functional disorders, but little is known about the pathophysiological effects of inflammation in the parathyroids. To date, this is the largest study of parathyroid tumors presenting with prominent lymphocytic infiltrates. Consistent with previous case reports and studies (4, 5), we observed two distinctly different patterns of inflammation. Since the inflammation was predominantly located in the tumor tissue, we propose that the infiltrates are tumor specific.

We identified 17 tumors with prominent, nodular germinal center-like inflammation based on CD45+ (scored as 2+). In the literature, only 15 such cases are reported, four of which have been obtained from autopsy series (4). While the histological impression of this inflammation was very distinct from the diffuse infiltrates, we were unable to identify an association to clinical variables or patients’ histories (autoimmune diseases were present in similar frequencies as in cases with diffuse inflammation). We hypothesized that parathyroid tumors might have a viral etiology and sought to comprehensively search for viral transcripts using transciptome sequencing. Viral transcripts were not detected in the investigated parathyroid tumors, suggesting that active viral transcription is not a common etiology for parathyroid tumors. However, given the limited number of tumors studied by transcriptome sequencing, we cannot rule out a selection bias of the selected cases with consequently false-negative results. Considering that the common tumor inducing viruses (Epstein-Barr virus, hepatitis B and C viruses, human papilloma virus, Kaposi’s sarcoma-associated herpesvirus and human T cell lymphotropic virus type 1) are all expressed at a considerable level in the associated tumor cells, a transcriptome approach is expected to detect similar virus sequences. Nevertheless, this study does not rule out untranscribed genomic viral sequences.

Our study mainly includes single adenomas, with very few patients presenting with multiglandular disease. Since we only selected cases with reported inflammation from our pathology archives, and no clear-cut case of multiglandular pHPT was detected, the occurrence of inflammation in multiglandular pHPT is expected to be low. At the same time, a putative finding of inflammation in multiple parathyroid tumors from the same patient at the same time point would argue in favor of a systemic inflammatory process. The lack of identified patients presenting with multiglandular pHPT in combination with the normalization of hypercalcemia in all cases where this information was available should argue against a systemic auto-immune reaction against parathyroid tissue. An extended series focusing specifically on multiglandular disease could potentially help us delineate this topic further. However, we cannot rule out that an autoimmune process could predominantly affect a parathyroid tumor population, whereby normal parathyroid glands could remain unaffected by inflammation, while the single adenoma is not.

We observed a significant association between presence of inflammatory infiltrates and increase in the patients’ serum levels of PTH, without a significant increase in tumor weight or evidence of degenerated tumor tissue. Several studies have shown that tumor weight is the main determinant of patients’ PTH and calcium levels (15, 21). Since inflammation did not influence tumor weight, the observed association could indicate a functional relationship between tumor-infiltrating CD8+ lymphocytes (which were the predominating cell type) and tumor PTH secretion. Given the composition of the inflammatory infiltrates, the observed association to higher PTH levels could be explained either by direct interaction between immune cells and parathyroid cells, changes in the local cytokine milieu affecting parathyroid tumor cells or an unknown confounding factor. An interaction between tumor and immune cells could be of value in the medical management of hyperparathyroidism. The one fourth of the patients who were prescribed anti-inflammatory medication at the time of surgery had significantly lower levels of serum ionized calcium. Following studies might clarify how different anti-inflammatory medication affects hyperparathyroidism in different patients and clinical settings.

Patients with inflammatory parathyroid tumors frequently had co-existing autoimmune disease (including Graves’ disease, diabetes mellitus type I, autoimmune hypothyroidism, Crohn’s disease or ulcerative colitis). Previous studies have investigated a putative autoimmune etiology for pHPT, including identification of antibodies targeting CASR in analogy with Graves’ disease (22). There have also been epidemiological studies associating pHPT to coeliac disease (23), autoimmune atrophic gastritis (24) and autoimmune thyroiditis (25). Additionally, there are a few case reports of PGA patients presenting with parathyroid tumors, but there is no mentioning of tumor inflammatory infiltrates in these tumors. (26, 27). Chronic lymphocytic thyroiditis and Hashimoto’s disease can generate oxyphilic metaplasia in thyroid follicular cells. Interestingly, we observed that oxyphilic adenomas were significantly associated to both inflammatory infiltrates and autoimmune disease. Furthermore, in some tumors with mixed cell the inflammation was predominantly found in areas with oxyphilic differentiation. While we often see oxyphilic adenomas without inflammation, these observations suggest a potential relationship between inflammatory infiltrates, autoimmunity and oxyphilic differentiation. In the majority of cases, the inflammation was clearly restrained to tumor tissue. If the infiltrating lymphocytes are indeed linked to an autoimmune process, our data indicate that it should be targeting antigens expressed at higher levels in the tumor than the normal parathyroid tissue.

This study was based on a retrospective analysis of parathyroid tumors with histologically evident inflammatory infiltrates at the time of diagnosis, which could explain the different observations made by earlier reports of inflammatory infiltrates in parathyroid tissue. Shi *et al*. studied 20 parathyroid adenomas and five biopsies from normal parathyroid glands, identifying increased levels of inflammatory cells in the tumor as compared to the normal glands. They describe varying levels of CD8+ tumor-infiltrating lymphocytes (ranging from ~2% to 20%) suggesting that such infiltrates are not that uncommon in parathyroid adenomas (5). While they report similar levels of lymphocytes in biopsies from normal glands, we rarely observed inflammatory engagement of corresponding normal tissue. Since our cohort was based on the histological identification of inflammation (55 identified tumors over a 10-year period), it is likely that our material represents cases with more much more prominent inflammation.

As part of the clinical diagnostics of parathyroid tumors, tumor cell proliferation is sometimes determined by Ki-67 index, and the WHO classification of endocrine tumors from 2004 demand vigilant follow-up for tumors with proliferation counts exceeding 5%. Since lymphocytes are histologically similar to parathyroid cells, proliferating leukocytes could potentially give raise to falsely high Ki-67 counting in cases with diffuse inflammation. Review of the pathology reports revealed that no cases were reported to have a Ki-67 index above 2%, indicating that this problem is limited. Even so, a dual staining technique with CD45 would exclude the possibility of falsely high Ki-67 counting.

In conclusion, histologically prominent inflammation in parathyroid tumors is rare. At least one fourth of patients with inflammatory parathyroid tumors had a concomitant autoimmune disease, indicative of a common denominator. Oxyphilic adenomas were significantly associated with an autoimmune disease and presence of inflammatory infiltrates. Nodular inflammation is histologically distinct, but showed little adverse clinical presentation. Lymphocyte infiltrates significantly correlated to higher levels of patient’s levels of PTH and could potentially indicate a cytokine mediated change in parathyroid tumor cell endocrine activity. For patients with inflamed tumors, prescription of anti-inflammatory drugs at the time of surgery was significantly associated with lower calcium levels. Finally, we were unable to find evidence supporting active viral transcription in the investigated cases.

## Supplementary Material

Supporting Table 1

Supporting Table 2

## Declaration of interest

The authors declare that there is no conflict of interest that could be perceived as prejudicing the impartiality of this study.

## Funding

This study was supported by the Swedish Cancer Foundation, the Swedish Research Council, the Cancer Society in Stockholm, Karolinska Institutet and Stockholm County Council.
