# Supplementary material for: Inflammatory infiltrates in parathyroid tumors
Source: Eur J Endocrinol. 2017 Aug 30;177(6):445–53. doi: 10.1530/EJE-17-0277 (PMC5642267; doi:10.1530/EJE-17-0277)
Supplement: Supporting Table 1 [file eje-177-445-t001.pdf]

Supplementary Table S1: Clinicopathological data for parathyroid tumors with inflammatory infiltrates

Tumor	Patient	Sex (M/F)	Age (years)	Diagnosis	Weight (mg)	Dominating cell type	Serum ionized calcium (mmol/L)	Serum intact PTH (ng/L)	Plasma phosphate (mmol/L)	Autoimmune disease	Anti-inflammatory medication
1	1	M	68	Adenoma	950	Mixed	1.37	128	0.92	Yes	Yes
2	2	M	81	Adenoma	760	Oxyphilic	1.47	145	1.00	No	Yes
3	3	F	29	Adenoma	2243	Oxyphilic	1.76	308	0.86	No	No
4	4	M	67	Adenoma	370	Oxyphilic	1.39	95	1.00	Yes	Yes
5	5	F	59	Adenoma	609	Chief	1.39	158	0.79	No	No
6	6	F	77	Adenoma	1347	Oxyphilic	1.48	163	0.72	No	No
7	7	F	58	Adenoma	656	Chief	1.41	110	1.00	Yes	No
8	8	F	68	Adenoma	469	Oxyphilic	1.51	155	1.20	No	No
9	9	F	69	Adenoma	565	Oxyphilic	1.43	96	0.92	No	No
10	10	F	39	Adenoma	690	Chief	1.38	115	0.80	No	Yes
11	11	F	41	Adenoma	213	Chief	1.54	170	n.a.	No	No
13	13	F	77	Adenoma	270	Chief	1.40	147	0.84	No	No
14	14	F	41	Adenoma	1313	Mixed	1.62	76	0.67	No	No
15	15	F	61	Adenoma	390	Chief	1.48	102	1.00	No	No
16	16	F	45	Adenoma	100	Chief	1.38	46	1.30	No	No
17	17	F	56	Adenoma	262	Chief	n.a.	n.a.	n.a.	No	Yes
18	18	F	66	Adenoma	1092	Oxyphilic	1.43	205	0.80	Yes	No
19	19	F	61	Adenoma	2412	Oxyphilic	n.a.	112	0.76	Yes	No
20	20	F	32	Adenoma	200	Chief	1.46	75	n.a.	No	No
21	21	F	61	Adenoma	1611	Chief	1.55	166	0.90	No	No
22	22	F	67	Adenoma	302	Chief	1.49	222	n.a.	No	No
23	23	F	57	Adenoma	650	Chief	1.51	62	n.a.	Yes	Yes
24	24	M	48	Adenoma	1030	Chief	1.48	148	0.82	No	No
25	25	F	45	Adenoma	448	Chief	1.60	221	0.85	No	No
26	26	F	84	Adenoma	657	Chief	1.42	214	0.83	No	No
27	27	F	63	Adenoma	1573	Oxyphilic	1.42	196	0.95	Yes	No
29	29	F	70	Adenoma	320	Mixed	1.35	105	n.a.	No	Yes
29	29	F	56	Adenoma	455	Oxyphilic	1.37	123	0.81	Yes	Yes
30	30	M	73	Adenoma	1185	Chief	1.84	342	n.a.	Yes	No
31	31	F	43	Adenoma	227	Chief	n.a.	n.a.	n.a.	Yes	Yes
32	32	M	65	Adenoma	316	Chief	1.40	130	0.8	No	No
33	33	M	76	Adenoma	178	Chief	1.35	110	n.a.	No	No
34	34	F	78	Adenoma	1016	Oxyphilic	1.49	123	n.a.	Yes	Yes
35	35	F	68	Adenoma	266	Oxyphilic	1.34	85	1.00	No	No
36	36	F	41	Adenoma	468	Mixed	1.43	113	0.79	No	No

37	37	M	55	Adenoma	528	Chief	1.37	104	0.94	No	No
38	38	F	67	Adenoma	769	Chief	1.48	208	n.a.	No	No
39	39	F	56	Adenoma	151	Mixed	1.39	83	n.a.	Yes	Yes
40	40	M	39	Adenoma	736	Chief	1.43	189	0.55	No	No
41	41	M	60	Adenoma	936	Chief	1.56	132	0.84	No	No
42	42	F	37	Adenoma	1123	Chief	1.50	87	1.00	No	No
43	43	M	70	Adenoma	330	Oxyphilic	1.47	85	0.60	No	No
44	44	F	45	Adenoma	150	Chief	1.40	84	0.81	No	Yes
44	44	F	64	Adenoma	355	Chief	n.a.	208	0.94	No	Yes
45	45	M	56	Adenoma	1780	Chief	n.a.	142	0.75	No	No
46	46	F	42	Adenoma	1337	Oxyphilic	1.43	257	n.a.	n.a.	n.a.
47	47	F	54	Adenoma	673	Chief	1.72	272	n.a.	n.a.	n.a.
48	48	M	61	Adenoma	400	Chief	1.70	167	n.a.	n.a.	n.a.
49	49	M	62	Adenoma	1900	Chief	1.44	103	n.a.	n.a.	n.a.
50	50	F	55	Adenoma	188	Chief	1.38	148	n.a.	n.a.	n.a.
51	51	F	55	Adenoma	373	Chief	1.38	83	n.a.	n.a.	n.a.
52	52	M	64	sHPT	n.a.	Hyperplasia	n.a.	972	0.72	Yes	No
53				sHPT	n.a.	Hyperplasia					
54	53	F	64	tHPT	n.a.	Hyperplasia	n.a.	n.a.	n.a.	n.a.	No
55				tHPT	n.a.	Hyperplasia					

M = Male; F = Female

n.a. = not available, sHPT = secondary hyperparathyroidism, tHPT = tertiary hyperparathyroidism
