# Supplementary material for: Inflammatory infiltrates in parathyroid tumors
Source: Eur J Endocrinol. 2017 Aug 30;177(6):445–53. doi: 10.1530/EJE-17-0277 (PMC5642267; doi:10.1530/EJE-17-0277)
Supplement: Supporting Table 2 [file eje-177-445-t002.pdf]

Supplementary Table S2: Immunohistochemical characterization using Cluster of Differentiation (CD) markers in parathyroid tumors

			CD4		CD8		CD20		CD45		
Tumor	Patient	Diagnosis	Diffuse	Nodular	Diffuse	Nodular	Diffuse	Nodular	Diffuse	Nodular	Normal rim
1	1	Adenoma	+0	+2	+2	+1	+0	+1	+1	+1	unaffected
2	2	Adenoma	n.i.	n.i.	+1	+0	+0	+1	+1	+0	unaffected
3	3	Adenoma	n.i.	n.i.	+1	+1	+0	+1	+2	+0	unaffected
4	4	Adenoma	n.i.	n.i.	+0	+2	+0	+2	+1	+2	n.a.
5	5	Adenoma	+1	+0	+1	+1	+0	+1	+1	+1	unaffected
6	6	Adenoma	+1	+0	+1	+1	+0	+1	+2	+1	n.a.
7	7	Adenoma	n.i.	n.i.	+0	+1	+0	+1	+0	+1	unaffected
8	8	Adenoma	n.i.	n.i.	+2	+0	+0	+1	+2	+0	unaffected
9	9	Adenoma	n.i.	n.i.	+2	+1	+0	+1	+1	+2	n.a.
10	10	Adenoma	+1	+0	+1	+0	+0	+0	+1	+1	n.a.
11	11	Adenoma	n.i.	n.i.	+1	+1	+0	+1	+2	+2	unaffected
13	13	Adenoma	+1	+1	+1	+1	+0	+2	+2	+2	unaffected
14	14	Adenoma	+0	+1	+0	+1	+0	+1	+1	+1	unaffected
15	15	Adenoma	n.i.	n.i.	+2	+0	+1	+1	+2	+0	n.a.
16	16	Adenoma	+2	+1	+2	+0	+0	+1	+2	+2	n.a.
17	17	Adenoma	+1	+1	+2	+1	+0	+1	+2	+2	n.a.
18	18	Adenoma	n.i.	n.i.	+1	+0	+0	+0	+1	+1	unaffected
19	19	Adenoma	n.i.	n.i.	+1	+0	n.a.	n.a.	+1	+0	n.a.
20	20	Adenoma	n.i.	n.i.	+2	+1	+0	+2	+2	+2	unaffected
21	21	Adenoma	n.i.	n.i.	+2	+1	+0	+2	+2	+2	n.a.
22	22	Adenoma	n.i.	n.i.	+1	+0	n.a.	n.a.	+2	+0	unaffected
23	23	Adenoma	n.i.	n.i.	+2	+1	+1	+1	+1	+1	unaffected
24	24	Adenoma	n.i.	n.i.	+1	+1	+0	+2	+1	+2	unaffected
25	25	Adenoma	+0	+1	+2	+2	+0	+2	+2	+2	n.a.
26	26	Adenoma	+0	+1	+1	+1	+0	+2	+0	+1	unaffected
27	27	Adenoma	n.i.	n.i.	+1	+1	+0	+2	+1	+2	n.a.
29	29	Adenoma	n.i.	n.i.	+1	+0	+0	+0	+1	+0	n.a.
29	29	Adenoma	n.i.	n.i.	+1	+1	+0	+0	+1	+1	unaffected
30	30	Adenoma	n.i.	n.i.	+2	+1	+0	+1	+1	+1	n.a.
31	31	Adenoma	n.i.	n.i.	+2	+0	+0	+0	n.a.	n.a.	n.a.
32	32	Adenoma	n.i.	n.i.	+1	+1	+0	+0	+1	+1	unaffected
33	33	Adenoma	n.i.	n.i.	+0	+1	+0	+1	+0	+1	n.a.
34	34	Adenoma	n.i.	n.i.	+1	+1	+0	+1	+1	+1	affected
35	35	Adenoma	n.i.	n.i.	+1	+1	+0	+1	+1	+1	n.a.
36	36	Adenoma	+1	+0	+1	+0	+0	+1	+1	+1	n.a.
37	37	Adenoma	n.i.	n.i.	+2	+0	+0	+1	+1	+1	affected

38	38	Adenoma	n.i.	n.i.	+1	+0	+0	+0	+1	+1	unaffected
39	39	Adenoma	n.i.	n.i.	+1	+0	+0	+0	+1	+0	n.a.
40	40	Adenoma	n.i.	n.i.	+1	+1	+0	+1	+1	+1	unaffected
41	41	Adenoma	n.i.	n.i.	+2	+0	+0	+0	+2	+0	n.a.
42	42	Adenoma	+1	+1	+2	+1	+1	+1	+1	+1	unaffected
43	43	Adenoma	+0	+1	+0	+1	+0	+1	+0	+1	unaffected
44	44	Adenoma	+0	+1	+1	+2	+0	+2	+1	+2	n.a.
44	44	Adenoma	+0	+1	+2	+0	+0	+2	+1	+2	n.a.
45	45	Adenoma	+0	+2	+0	+2	+0	+2	+1	+2	n.a.
46	46	Adenoma	n.i.	n.i.	+1	+0	+0	+0	+2	+1	unaffected
47	47	Adenoma	n.i.	n.i.	+1	+0	+0	+0	+1	+1	unaffected
48	48	Adenoma	n.i.	n.i.	+1	+0	+0	+1	n.a.	n.a.	unaffected
49	49	Adenoma	+0	+2	+0	+2	+0	+2	+0	+2	unaffected
50	50	Adenoma	+0	+2	+0	+2	+0	+2	+0	+2	unaffected
51	51	Adenoma	+0	+1	+0	+1	+0	+1	+0	+2	n.a.
52	52	sHPT	n.i.	n.i.	+2	+1	+0	+1	+1	+1	n.a.
53		sHPT	n.i.	n.i.	+1	+1	+0	+0	+1	+1	n.a.
54	53	tHPT	+0	+1	+2	+0	+0	+2	+2	+2	n.a.
55		tHPT	n.i.	n.i.	+2	+0	+0	+2	+2	+2	n.a.

n.a. = not available, n.i. = non informative (i.e. high background in CD4 immunostaining),

sHPT = secondary hyperparathyroidism, tHPT = tertiary hyperparathyroidism
